# Prevalence and Risk Factors of Hypoglycemia Unawareness Among Patients With Type 1 and Type 2 Diabetes Mellitus in Saudi Arabia: A Systematic Review and Meta-Analysis

**DOI:** 10.7759/cureus.92614

**Published:** 2025-09-18

**Authors:** Mohammed A Almohammadi, Adeeb M Aloufi, Azzam M Alharbi, Abdullah F Aljohani, Osama A Almohammadi, Anas A Almohammadi, Waleed H Alharbi, Faisal A Almutairi, Abdullah F Almohammadi, Ahmed S Alhuwayfi

**Affiliations:** 1 Endocrinology, Diabetes, and Metabolism, Taibah University, Medina, SAU; 2 Family Medicine and Diabetology, Diabetes Center, King Salman Bin Abdulaziz Medical City, Medina, SAU

**Keywords:** diabetes mellitus, hypoglycemia unawareness, impaired awareness of hypoglycemia, prevalence, risk factors, saudi arabia

## Abstract

Objective: Hypoglycemia unawareness (HU), also known as impaired awareness of hypoglycemia (IAH), is a significant complication of insulin therapy that increases the risk of severe neuroglycopenic events in patients with type 1 diabetes mellitus (T1DM) and type 2 diabetes mellitus (T2DM). Research data from Saudi Arabia on this topic is limited and inconsistent. This systematic review and meta-analysis aimed to identify and summarize studies that report the prevalence, risk factors, and clinical outcomes of HU among individuals with T1DM and T2DM in Saudi Arabia.

Methods: Following the Preferred Reporting Items for Systematic Reviews and Meta-Analyses (PRISMA) guidelines, a comprehensive search was conducted across PubMed, Scopus, Web of Science, Cochrane Library, and Google Scholar up to July 2025 for observational studies that reported the prevalence or risk factors of HU in Saudi adults with diabetes. A total of 11 cross-sectional studies (n = 4,171) met the inclusion criteria. The quality of these studies was appraised using tools from the Joanna Briggs Institute (JBI). Proportions were pooled using a random-effects model (OpenMetaAnalyst), and heterogeneity was assessed using the I-squared (I²) statistic. Leave-one-out sensitivity analyses were performed to evaluate the robustness of the findings.

Results: The overall pooled prevalence of HU was found to be 41.9% (95% confidence interval [CI]: 21.2-62.6%), with high heterogeneity (I² = 99.7%, p <0.001). Sensitivity analyses indicated minimal fluctuation in the prevalence estimates (ranging from 36.8% to 45.5%) when excluding any single study, confirming the stability of the pooled estimate. Key risk factors identified included poor diabetes knowledge, specifically a lack of awareness regarding insulin type, dosage, and the most recent glycated hemoglobin (HbA1c) levels, insufficient medical follow-up, and macrovascular complications (such as prior stroke and ischemic heart disease). It was also noted that lower daily insulin doses were associated with HU.

Conclusion: Nearly half of insulin-treated diabetes patients in Saudi Arabia display impaired awareness of hypoglycemia. Addressing modifiable factors, particularly inadequate patient education and follow-up, is crucial. Implementing standardized screening protocols for HU and structured diabetes self-management education is essential, as these measures may help reduce the risk of severe hypoglycemic events in this population.

## Introduction and background

Diabetes mellitus (DM) is a growing global health concern, particularly in low- and middle-income countries. In Saudi Arabia, the prevalence of DM among adults aged 20 to 79 years is estimated at 18.3%, with approximately 4.27 million adults affected in 2024 [1]. As the burden of DM rises, so does the occurrence of its complications, one of the most critical being hypoglycemia unawareness (HU).

Hypoglycemia unawareness, also referred to as impaired awareness of hypoglycemia (IAH), is a condition where individuals with DM fail to recognize the early neurogenic symptoms of low blood glucose, such as palpitations, tremors, or sweating [2]. This failure in symptom recognition increases the risk of severe hypoglycemic episodes, which can lead to seizures, loss of consciousness, hospitalization, or even sudden death [3]. It is especially common in insulin-treated patients, both in type 1 diabetes mellitus (T1DM) and advanced type 2 diabetes mellitus (T2DM), and is often associated with long-standing DM, tight glycemic control, and frequent hypoglycemic episodes [4].

International studies indicate that up to 40% of insulin-treated individuals with DM experience IAH, which is associated with a more than six-fold increased risk of severe hypoglycemic episodes [5]. Despite its clinical importance, awareness and reporting of HU in Saudi Arabia remain limited. Cultural, educational, and healthcare system-related factors may influence how hypoglycemia is perceived, managed, and reported in the Saudi population [6]. Therefore, understanding the prevalence and associated risk factors of HU in Saudi Arabia is essential for guiding local healthcare providers, improving diabetes education, and enhancing patient safety.

This systematic review aims to synthesize existing literature on HU among patients with DM in Saudi Arabia, estimating its prevalence and identifying the associated risk factors. The findings will offer valuable insights to inform preventive strategies, risk stratification, and tailored patient management in the Saudi context.

## Review

Methodology

This systematic review was performed following the Preferred Reporting Items for Systematic Reviews and Meta-Analyses (PRISMA) guidelines [7]. The review protocol was prospectively registered in the International Prospective Register of Systematic Reviews (PROSPERO) (registration no. CRD420251090899). A comprehensive literature search was conducted across five databases: PubMed, Scopus, Web of Science, Cochrane Library, and Google Scholar, with the final search taking place in July 2025. The search strategy was designed to effectively combine medical subject headings (MeSH) and free-text terms using Boolean operators. The resulting search string incorporated various relevant terms, including ("diabet" OR "diabetes mellitus" OR "T1DM" OR "T2DM" OR "type 1 diabetes" OR "type 2 diabetes") AND ("hypoglycemia unawareness" OR "impaired awareness of hypoglycemia" OR "IAH" OR "hypoglyc") AND ("prevalence" OR "incidence" OR "epidemiol" OR "risk factor" OR "predictor" OR "associated factor") AND ("Saudi Arabia" OR "KSA").

Inclusion and exclusion criteria

Studies were deemed eligible for inclusion if they were conducted in Saudi Arabia and reported on the prevalence and/or risk factors associated with HU in patients with either T1DM or T2DM. Only observational designs, such as cross-sectional and cohort studies, were considered. No restrictions were applied based on publication year; however, all studies that met the eligibility criteria were published between 2020 and 2025. The detailed inclusion and exclusion criteria are summarized in Table 1.

**Table 1:** Inclusion and exclusion criteria for study selection HU: Hypoglycemia unawareness

Criteria Type	Specific criterion
Inclusion criteria	Conducted in Saudi Arabia
	Focused on patients with T1DM or T2DM
	Reported prevalence and/or risk factors of HU
	Observational study design (cross-sectional or cohort)
	Published in English
Exclusion criteria	Conducted outside Saudi Arabia
	Did not specifically address HU
	Non-observational designs (e.g., reviews, editorials, opinion pieces, case reports)
	Full text inaccessible

Selection of articles and data extraction

The study selection process was conducted independently by three reviewers utilizing Rayyan (Qatar Computing Research Institute (QCRI), Doha, QAT), an online screening platform, with any discrepancies resolved through discussions or consultation with a senior reviewer. For data collection, three independent reviewers extracted essential information using a standardized form, which encompassed key characteristics of the studies: participant demographics, clinical variables, definitions, prevalence of HU, associated risk factors, and outcomes. A total of predefined variables was compiled, and any disagreements among reviewers were addressed through collaborative discussions. The collected data included a comprehensive array of aspects such as the study title, article ID, study design, publication year, regional settings in Saudi Arabia, total sample size, mean age, gender distribution, BMI, socioeconomic status, smoking and alcohol use, comorbidities (like hypertension and cardiovascular diseases), type and duration of diabetes mellitus, glycated hemoglobin (HbA1c) or blood glucose levels, treatment methods, inclusion and exclusion criteria, definitions of HU, frequency of hypoglycemic events, neurogenic versus neuroglycopenic symptom profiles, hospitalization requirements, timing and setting of episodes, assessment tools for HU, knowledge and attitude scores, severity, risk factors, significant findings (p<0.05), study limitations, and overall conclusions.

Quality assessment

Each study included in the review was evaluated using the Joanna Briggs Institute (JBI) Critical Appraisal Tools [8] tailored to the specific design of each research. We utilized the Checklist for Analytical Cross-Sectional Studies along with the Checklist for Prevalence Studies to ensure a thorough assessment. The checklist prompts reviewers to ask key questions regarding the appropriateness of the sampling frame, the adequacy of the sample size, and the validity of the condition identification methods. This structured inquiry covers study design, sampling strategy, measurement reliability, and statistical analysis. Examining data collection standardization and response rates ensures that reported prevalence estimates are valid and generalizable. Additionally, it encourages consideration of confounding factors and appropriate statistical techniques, which enhances transparency in systematic review decisions, ultimately improving the reliability of evidence-based healthcare recommendations and informing policy and clinical guidelines. The evaluations were carried out by two independent reviewers, who collaborated to resolve any discrepancies through constructive discussion, thereby enhancing the reliability of the appraisals.

Statistical analysis

Data were entered and analyzed using OpenMetaAnalyst [9]. Dichotomous data were analyzed as proportions and a 95% confidence interval (CI). Statistical heterogeneity among the studies was assessed using I-squared (I²), and statistical significance was set at a p-value < 0.05. Leave-one-out sensitivity analysis was done for high heterogeneity.

Results

**Figure 1:**
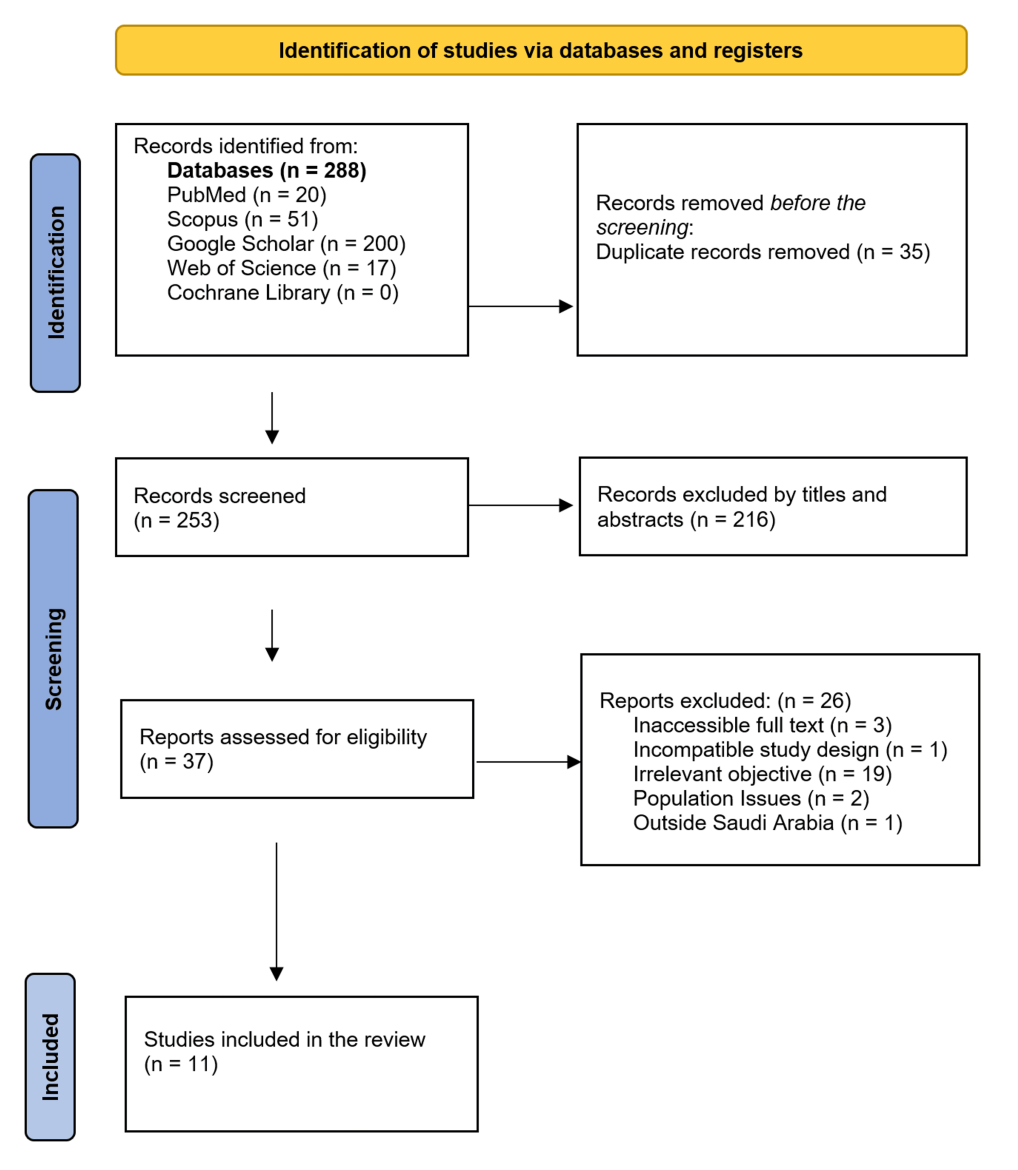
PRISMA flow diagram of the criteria for selecting studies in the systematic review PRISMA: Preferred Reporting Items for Systematic Reviews and Meta-Analyses

The initial search yielded a total of 288 records, categorized as follows: 200 from Google Scholar, 51 from Scopus, 20 from PubMed, and 17 from Web of Science. After eliminating duplicates, 253 records were retained. A screening of titles and abstracts for all 253 studies resulted in a review of 37 full-text articles. Ultimately, 11 studies met the eligibility criteria and were included in the final evaluation (Figure 1).

Table 2 shows the characteristics of 11 cross-sectional studies conducted from 2020 to 2024, focusing on HU among 4,869 patients with T1DM and T2DM in various regions of Saudi Arabia. The prevalence of hypoglycemic events was found to be significant and varied, with reported rates ranging from 19.2% for severe events to 57.4% of participants experiencing at least one incident in the prior three to 12 months. Weekly episodes affected between 26% and 41.4% of individuals. The assessment of HU primarily relied on validated tools, with common methods including the Clarke score and the Gold method, alongside knowledge-based questionnaires and self-reported awareness surveys. Symptoms associated with hypoglycemia showed a predominance of neurogenic manifestations, such as sweating and palpitations, while neuroglycopenic symptoms were also frequent and severe, including drowsiness, dizziness, confusion, and seizures. Significant risk factors for HU and hypoglycemia included a longer duration of diabetes, intensive insulin therapy, and lower socioeconomic status, characterized by low income and educational attainment. There were notable deficits in knowledge and awareness of HU among participants, with only about half reporting "good knowledge" of the condition. Methodological limitations, such as inconsistent definitions and assessment tools, hindered direct comparisons across studies. These findings highlight HU as a significant clinical issue in Saudi Arabia, influenced by disease-related factors, treatment intensity, socioeconomic challenges, and gaps in knowledge, indicating a need for standardized assessments and targeted interventions.

**Table 2:** Study characteristics for prevalence and risk factors of hypoglycemia unawareness among patients with T1DM and T2DM in Saudi Arabia CI: Confidence interval; Clarke: Clarke hypoglycemia awareness score; DM: Diabetes mellitus; HbA1c: Glycated hemoglobin; HU: Hypoglycemia unawareness; IAH: Impaired awareness of hypoglycemia; IQR: Interquartile range; LOC: Loss of consciousness; M/F: Male/female; OHA: Oral hypoglycemic agents; SAR: Saudi Riyal; SMBG: Self-monitoring of blood glucose; T1DM: Type 1 diabetes mellitus; T2DM: Type 2 diabetes mellitus; HA: Hypoglycemia awareness

First author, year	Study design and setting	Age	M/F	Socioeconomic status	Type and duration of DM	Treatment for DM	HU definition/assessment tool and scoring	Number of hypoglycemic events in the past six to 12 months	Neurogenic vs. neuroglycopenic symptom profiles
Alqahtani et al., 2024 [10]	Cross-sectional study in Family Medicine clinics at King Fahad Armed Forces Hospital in Aseer, Saudi Arabia.	18-60 years	93/142	Income: <3,000 SAR 52.3%; 3,000–4,999 13.2%; 5,000–9,999 17.4%; 10,000–14,000 13.2%; ≥15,000 3.8%. Education: none 27.2%; primary 17.9%; intermediate 17.4%; high school 26.4%; university+ 11.1%.	T2DM. Duration not reported	Oral hypoglycemic agents were prescribed to 63.0% of patients, and 21.7% were on insulin therapy.	A validated questionnaire by Alanazi et al. assessed patients’ awareness of hypoglycemia attacks; scoring ≥75% (12 of 16 points) was considered good knowledge and practice.	50.6% reported ≥1 hypoglycemic attack	Palpitations, tremors, drowsiness
Alhedhod et al., 2024 [6]	Cross-sectional study at the Endocrine and Diabetes Center in Al-Ahsa, Saudi Arabia.	Mean age: 44.6	201/189	Not reported	T1DM: 41.3% T2DM: 58.7% Time since diagnosis: One to five years 15.9%, five to 10 years 25.1%, 10–15 years 16.4%, 15–20 years 14.1%, >20 years 28.5%	Insulin injections: 98.5%, Insulin pump: 1.5%	Clarke score (eight items, range 0–7). Impaired awareness if ≥4; normal ≤2; 3 = undetermined	Not reported	Not reported
Oraibi et al., 2024 [11]	Cross-sectional study at Jazan Endocrine and Diabetes Center, Saudi Arabia.	13-65 years	67/84	Income: <5000 SAR (31.8%), 5000–9999 SAR (37.7%), 10000–14999 SAR (18.5%), ≥15000 SAR (11.9%). Education: Illiterate (11.9%), High school or less (48.3%), Diploma/Bachelor (39.7%)	T1DM. ≥10 years: 55.0%, <10 years: 45.0%	Basal bolus (78.1%), Mixed insulin (19.2%), Insulin pump (2.6%)	Blood glucose <70 mg/dL; Clarke score ≥4 indicates IAH	19.2% had ≥1 severe event in the past year	Shaking (28.5%), sweating (24.8%), drowsiness (23.1%), speech difficulty (21.7%), headache (38.9%)
Al Hussaini et al., 2023 [12]	Cross-sectional online survey-based study in Al-Ahsa, Saudi Arabia.	Five-65	132/106	Education: Higher (59.7%), High school (18.5%), Intermediate (8.8%), Primary (8.8%), No formal education (4.2%)	T1DM 46.6%, T2DM 51.3%, unsure 2.1%; duration not reported	Insulin only 49.2%, insulin + other meds 50.8%	Hypoglycemia <70 mg/dL (ADA) plus 13-item questionnaire: >7 good, 6–7 fair, <6 poor.	Not reported	Not reported
Surrati et al., 2023 [13]	A cross-sectional study was conducted at the Diabetes Center and four PHCs across Madinah city, Saudi Arabia.	≥14 years	164/249	Not reported	T2DM 68.5% (disease duration 15 years, insulin use five years); T1DM 31.5% (disease duration 10 years, insulin use 10 years)	The majority of participants (60.8%) were managed with insulin alone, while 39.2% received a combination of insulin and oral hypoglycemic agents (OHAs).	Clarke’s method classified individuals as having HU if their score was ≥4, while the modified Pedersen-Bjergaard method employed a 3-point scale (always/sometimes/never) to evaluate symptom recognition.	Not reported	Not reported
AlTowayan et al., 2023 [14]	A cross-sectional study through an online questionnaire based in Al-Qassim, Saudi Arabia.	Mean age is 35.9 ± 13.0	62/151	Education: University 73.7%, Secondary or less 26.3%	All participants had T2DM with no reported disease duration	OHA and/or insulin	IAH was assessed using two standardized tools: the Clarke and Gold methods. In both instruments, a score of ≥4 was used to classify individuals as having IAH.	Symptomatic: One to three months 26.8%; weekly 24.9%; Asymptomatic: One to three months 23%, weekly 19.7%	Weakness 51.2%, disorientation 30.5%; Severe outcomes: unconsciousness 10.3%, seizures (past 12 months) 21.1%
Alreshidi et al., 2023 [15]	A cross-sectional study through an online questionnaire based in Hail, Saudi Arabia.	20–60 years	289/286	Income: <5,000 SAR 39.3%, unemployed 19.1%; Education: Bachelor’s 51.7%, Elementary/high school 34.3%, no education 5.9%, Postgraduate 8.2%.	All T2DM; disease duration: <5 years 43.5%, five–10 years 30.6%, >10 years 25.9%.	Diet 25%, oral meds 34.3%, insulin only 21.7%, oral + insulin 19%	A self-reported awareness survey was used to assess awareness among participants.	Past three months: ≥1 episode 48.2%; of these, <3 episodes 73.7%, three to six episodes 21%	Dizziness (age 20–30) 84.1%; fatigue 73.2%; palpitations 58.4%
Hassounah et al., 2022 [16]	A cross-sectional study in the Diabetes Treatment Center, Prince Sultan Military Medical City, Riyadh, Saudi Arabia.	No explicit age range was stated	97/145	61.2% reported having completed school-level education, while 38.8% had attained college-level qualifications	All participants had T1DM. 47.5% had been diagnosed for less than 10 years, while 52.5% had lived with the condition for 10 years or more.	All participants reported receiving insulin therapy for less than a year.	IAH was assessed using the Gold method, with a score of ≥4 indicating IAH	57.4% of participants had more than one hypoglycemic event during the study period	Edinburgh scale: Most symptoms ≥4 except confusion; females: warmth, pounding heart, concentration issues; married: drowsiness, dizziness; HbA1c >7%: drowsiness, hunger; ≥10 y diabetes: dizziness, warmth; smokers: nausea
Alanazi et al., 2021 [17]	A cross-sectional study through an online survey in Al-Jouf, Saudi Arabia.	Over 18 years	130/143	Income: <5,000 SAR 70.9%, ≥15,000 SAR 1.8%; Education: University 57.5%, Secondary or less 28.6%, other 13.9%.	T1DM and T2DM with no reported disease duration	More than half of the participants (55.3%) were treated with insulin alone, while 42.9% used oral hypoglycemic agents. A small proportion (1.8%) received both insulin and oral medications.	Awareness of hypoglycemia was assessed using a custom 16-item validated questionnaire, with scores ≥12 indicating good awareness.	41.4% reported experiencing episodes once per week, 26% had three to six episodes weekly, and 2.9% experienced more than six episodes per week. Notably, 22.7% reported no hypoglycemic episodes.	Dizziness 36.6%, drowsiness 32.6%, sweating 30%; loss of consciousness 29.3%; hunger/weakness 27.5%; shaking 24.2%; palpitations/tremors 17.9%; vision/speech issues 8.8%
Al Zahrani et al., 2021 [18]	A cross-sectional study at National Guard PHCs, Jeddah, Saudi Arabia.	24–88 years	153/208	Income: <5,000 SAR 35.2%, 5,000–10,000 SAR 39.1%, >15,000 SAR 2.5%, no income 10.2%; Education: Literate 34.1%, elementary/high school 20.8%, intermediate 15.8%, university 8.0%	T2DM 94.5%, T1DM 5.5%; diabetes duration median 10 years (IQR 5–15)	The majority of the participants (86.4%) were treated with oral hypoglycemic agents, while 39.1% received Insulin treatment.	A newly developed 42-item questionnaire was used to assess HA knowledge, where score ≥31.5 = good; 21–31.5 = moderate; <21 = poor knowledge	30.8% of participants had prior hypoglycemia experience. Almost half of the participants had hypoglycemia in the past month, with 8.2% requiring hospitalization.	Fatigue 69.5%, shakiness 47.1%, sweating 44.3%, palpitations 41.3%, blurred vision 39.3%, cold extremities 20.8%, loss of consciousness 20.5%, confusion 16.1%, nervousness 14.1%.
Murad et al., 2020 [19]	A nationwide analytical cross-sectional study through an online survey across Saudi Arabia.	20–76 years	486/594	Education: Students 28.7%, High School 27.8%, University 25%, no schooling 18.5%; income data not available.	32.4% had T1DM and 67.6% had T2DM. The mean duration of diabetes was 11 years, with 46.4% living with the condition for more than 15 years.	Not reported	IAH was classified using two validated methods: the Gold questionnaire, where a score >4 indicated impaired awareness, and the Pedersen-Bjergaard method, where any response other than “always” to the awareness question indicated IAH.	Hypoglycemia symptoms: Monthly 33.3%, weekly 16.7%, twice weekly 16.7%, infrequent 33.3%.	Reported symptoms included palpitations, fainting, confusion, and loss of consciousness (LOC). However, no detailed breakdown by symptom type was available.

Table 3 shows a significant and variable burden of HU among diabetic patients in Saudi Arabia, with prevalence rates varying widely due to differences in assessment methods. The study consistently found pervasive deficits in hypoglycemia awareness and knowledge, with 56% to 92.2% of participants demonstrating a poor to moderate understanding of symptoms, prevention, and management of hypoglycemia. Key risk factors contributing to HU include a longer duration of diabetes (10 years or more), a diagnosis of T2DM, older age (over 65 years), female gender, lower socioeconomic status (such as low income, unemployment, and limited education), macrovascular complications, and inadequate disease management practices, including infrequent glucose monitoring and poor clinical follow-up. Additionally, critical knowledge gaps exacerbate these risks, with only one-third of patients understanding glucagon use or recognizing asymptomatic hypoglycemia. There are also widespread misconceptions about complications and suboptimal acute management, particularly the tendency to rely on sweets or fruit juice instead of fast-acting carbohydrates. These factors contribute to severe outcomes, including high rates of unconsciousness (10.3% to 29.3%) and seizures (21.1%). The findings underscore the urgent need for standardized screening for HU, culturally tailored education programs targeting high-risk groups, and the systematic integration of structured hypoglycemia education. This education should focus on symptom recognition, preventive strategies, and evidence-based acute interventions to mitigate preventable complications.

**Table 3:** Outcomes of prevalence and risk factors of HU among patients with T1DM and T2DM in Saudi Arabia HU: Hypoglycemia unawareness; BMI: Body mass index; CI: Confidence interval; Clarke: Clarke hypoglycemia awareness score; DM: Diabetes mellitus; HbA1c: Glycated hemoglobin; ; IAH: Impaired awareness of hypoglycemia; LOC: Loss of consciousness; OHA: Oral hypoglycemic agents; OR: Odds ratio; SAR: Saudi Riyal; SMBG: Self-monitoring of blood glucose; T1DM: Type 1 diabetes mellitus; T2DM: Type 2 diabetes mellitus

First author, year	HU awareness and knowledge score	Significant risk factors (p < 0.05)	Key findings	Conclusion
Alqahtani et al., 2024 [10]	Poor awareness dominates this cohort, with 87.1% (205/235) of participants unable to recognize or appropriately respond to hypoglycemic signs.	Gender, age, occupation	Poor awareness was reported in 87.1%. Main triggers were skipping meals/fasting (63.7%) and hypoglycemic drugs (39.5%). Over half considered hypoglycemia life-threatening (55.3%) and likely to cause severe complications (55.7%). In acute events, 74.4% used sweets and 60.5% fruit juice, while only 24.8% relied on frequent glucose monitoring as prevention.	Poor awareness was common, affecting 87.1% of participants who could not recognize early symptoms. Significant links with gender, age, and occupation highlight the need for tailored education. Targeted interventions combining symptom-recognition training, reminders for self-monitoring, and culturally appropriate materials are essential to reduce severe episodes and improve safety.
Alhedhod et al., 2024 [6]	Impaired awareness: 23.8% (93/390) Normal awareness: 76.2%	Type of diabetes (T2DM vs T1DM), p = 0.038	23.8% of patients had impaired hypoglycemia awareness. T2DM patients are more likely to have IAH than T1DM (P = 0.038). No significant associations with age, age at diagnosis, HbA1c, duration since diagnosis, retinal exam, insulin modality, or nephropathy screening. Mean Clarke scores: T1DM 9.80 ± 1.3 vs T2DM 9.42 ± 1.3 (P = 0.005).	Nearly one-quarter of insulin-treated diabetics exhibited IAH, especially those with T2DM. Broader screening tools and additional risk-factor analyses are recommended.
Oraibi et al., 2024 [11]	25.2% (38/151) classified as impaired awareness (≥4)	BMI ≥25 (OR = 2.99), employed (OR = 18.2), DM duration ≥10 yrs (OR = 3.96), income ≥15000 SAR (OR = 0.10), frequent monitoring protective	25.2% IAH prevalence; associated with BMI, occupation, and monitoring method	IAH was prevalent among Type Ⅰ DM patients. The associated factors included Age, BMI, income, occupation, duration of DM, glucose monitoring method, and frequency. The study recommends frequent checks of patients’ behaviors and regular screening for IAH as part of the disease’s follow-up.
Al Hussaini et al., 2023 [12]	The vast majority of participants (87.0%) reported good awareness scores, with only 6.7% (16/238) indicating fair scores and 6.3% (15/238) reporting poor scores.	Hypertension showed a notable link (p = 0.016), also receiving guidance from doctors (p < 0.001), exposure to social media (p = 0.027), and educational materials such as pamphlets or booklets (p = 0.031) were all significantly associated with awareness and risk levels.	Knowledge gaps were evident in hypoglycemia management. Only 34.9% understood glucagon use, showing poor emergency preparedness. Awareness of asymptomatic hypoglycemia was low (46.6%), raising the risk of unnoticed episodes. Gaps were also seen in home management (43.3%), fasting implications (43.7%), and driving-related risks (42%).	Physician-delivered education and certain media significantly boosted knowledge. Participants demonstrated high baseline knowledge, yet critical deficits remain in specific management domains. Regular, multimodal educational interventions, especially physician-led and supplemented by social media/pamphlets, are needed to sustain and deepen patient competence.
Surrati et al., 2023 [13]	Clarke’s tool identified HU in 25.2% (104/413) of participants, while the modified Pedersen-Bjergaard scale yielded a higher prevalence of 48.9% (202/413), including 7% (29/413) classified as severely impaired.	Several factors were linked to higher HU risk: poor knowledge of insulin type (p = 0.001), dose (p = 0.001), and latest HbA1c (p = 0.002). Clinical and behavioral contributors included poor medical follow-up (p = 0.003), prior stroke (p = 0.003), and ischemic heart disease (p = 0.048). A lower insulin dose (p = 0.030) was also a significant predictor.	HU was identified in 25.2% of participants by Clarke’s method and 48.9% by the modified Pedersen-Bjergaard scale, showing variation by tool. Clarke’s method found no difference between T1DM and T2DM, while the Pedersen-Bjergaard scale showed slightly higher prevalence in T2DM (50.5% vs. 45.4%, p = 0.038). HU was not linked to age, gender, diabetes duration, insulin use, HbA1c, SMBG, or microvascular complications. Poor diabetes knowledge and macrovascular complications (stroke, ischemic heart disease) were significant predictors.	HU remains prevalent despite treatment advances. Poor diabetes knowledge and inadequate follow-up are key modifiable risk factors. Structured education and regular HU screening are essential to reduce risk.
AlTowayan et al., 2023 [14]	IAH was identified in over half of the participants using both assessment tools. 52.1% (111/213) by Clarke’s method and 53.5% (114/213) by the Gold method.	No statistically significant risk factors were reported.	Weakness was the most common symptom. Notably, 34.7% reported symptoms at glucose >70 mg/dL, suggesting misattribution or poor recognition. No significant associations were found between IAH and demographic or clinical factors. Symptom frequency was low, with many experiencing episodes only once every six months or less.	Patients with T2DM in the Al Qassim region showed insufficient knowledge of hypoglycemia, reflected by the high prevalence of IAH. This highlights the urgent need for targeted education and structured awareness programs to improve recognition, reduce misattribution, and strengthen self-management.
Alreshidi et al., 2023 [15]	Only 56% (322/575) of participants demonstrated clear awareness of hypoglycemia symptoms, while 35.3% (204/575) were somewhat aware and 8.7% (50/575) lacked awareness entirely.	Significant association between the number of episodes and age group (p = 0.023)	Nearly half reported hypoglycemic episodes in the past three months, with dizziness, fatigue, and palpitations most common. Overall awareness was modest, with age-related differences in recognition. No validated awareness tool was used; the assessment relied only on self-reported symptoms.	Hypoglycemia is common yet under-recognized in non-insulin-treated T2DM patients. Poor symptom recognition in this group highlights the need for targeted education and structured follow-up to improve awareness, ensure timely intervention, and reduce risks.
Hassounah et al., 2022 [16]	IAH was identified in 62.8% (152/242) of participants	A longer duration of diabetes (≥10 years) was significantly associated with IAH (p = 0.019).	High prevalence of IAH was found among T1DM. No significant associations were found between IAH and age, gender, BMI, HbA1c, or smoking status.	IAH is highly prevalent in Saudi T1DM patients, with longer diabetes duration as a key risk factor. Symptom recognition varied by gender, marital status, education, HbA1c, and smoking. These findings stress the need for structured education and proactive screening to reduce complications and improve safety.
Alanazi et al., 2021 [17]	Only 37.4% of participants demonstrated good awareness of hypoglycemia, while the majority (62.6%) (171/273) had poor awareness.	IAH was significantly associated with several sociodemographic factors, including gender (p = 0.018), age (p = 0.015), education level (p < 0.001), marital status (p < 0.001), and monthly income (p < 0.001).	Prior hypoglycemia was reported by 49.1%, and 72.5% knew the term “hypoglycemia.” Knowledge sources were mainly family/friends (42.5%) and media (41.8%). Many viewed hypoglycemia as serious, with 64.8% calling it life-threatening and 70.3% linking it to severe complications. For management, fruit juice (67.8%) and sweets (39.2%) were the most used and most commonly recognized treatments. Preventive strategies included consumption of sugar-rich foods (51.3%) and adherence to prescribed medications and dietary plans (46.9%).	Awareness levels were suboptimal, with significant associations to gender, age, education, marital status, and income. Educational interventions tailored to demographic profiles are needed to improve recognition of hypoglycemia and management.
Al Zahrani et al., 2021 [18]	The mean knowledge score regarding hypoglycemia was 32.0 ± 8.2, yet a striking 92.2% (333/361) of participants fell into the poor knowledge category. Only 5.3% (19/361) demonstrated moderate understanding, and a mere 2.5% achieved scores indicative of good knowledge, underscoring a substantial educational gap.	Several demographic and clinical factors were significantly associated with higher knowledge scores, including male sex, younger age, university education, single status, student status, high income, T1DM, and prior experience with hypoglycemia (all p-values ≤ 0.001).	Although overall knowledge was low, 66.8% identified the correct treatment (15g fast-acting carbs). Preventive strategy awareness was lacking in 63.4%. Main causes cited were skipping meals (59.8%) and medication overdose (37.4%). Reported complications included coma (47.4%) and glaucoma (43.2%). Physicians were the main information source (68.1%).	Most patients had poor knowledge of hypoglycemia. Education should be tailored to demographic and clinical profiles, with emphasis on prevention, symptom recognition, and proper management strategies.
Murad et al., 2020 [19]	Based on the Gold method, 14.8% (160/1080) of participants were classified as having IAH, while the Pedersen-Bjergaard method identified a slightly higher prevalence of 20%.	IAH was more likely in individuals >65 y (p = 0.04), females (p = 0.04), and those with diabetes >15 y (p = 0.04). Lack of formal education (p = 0.03), residence in the Western region (p = 0.04), and T1DM (p = 0.04) were also significant risk factors.	IAH prevalence ranged from 14.8% to 20% by assessment method. Higher rates occurred in older adults, females, longer diabetes duration, lower education, and Western region residents. T1DM patients were more affected than T2DM patients. No participants reported daily symptoms, and 66.7% had none, highlighting the silent nature of IAH and the need for proactive screening.	IAH is a significant and under-recognized issue in Saudi diabetic patients. Prevalence varies by assessment method. Targeted screening and education are needed, especially for older, female, and less-educated populations with long-standing diabetes.

Table 4 shows the methodological appraisal identified strengths in core study execution, including adequate sample sizes, detailed subject descriptions, sufficient analytical coverage, standardized measurement of HU, and appropriate statistical methods. However, significant limitations undermine validity and generalizability, as nearly all studies had an inappropriate sample frame (10/11) and most used inadequate sampling methods (9/11). Additionally, six studies employed invalid or unclear HU identification methods, risking misclassification, and all studies failed to report or manage response rates, potentially introducing non-response bias. These flaws necessitate cautious interpretation of prevalence estimates and limit the generalizability of findings across Saudi Arabia's diabetic population.

**Table 4:** Critical appraisal of included studies using Joanna Briggs Institute (JBI) tools Joanna Briggs Institute (JBI) tools [8]

Study	Q1	Q2	Q3	Q4	Q5	Q6	Q7	Q8	Q9
Alhedhod et al., 2024 [6]	No	No	Yes	Yes	Yes	Yes	Yes	Yes	Unclear
AlTowayan et al., 2023 [14]	No	Unclear	Yes	Yes	Yes	Yes	Yes	Yes	No
Alqahtani et al., 2024 [10]	No	No	Yes	Yes	Yes	No	Unclear	Yes	Unclear
Alanazi et al., 2021 [17]	No	No	Yes	Yes	Yes	No	Unclear	Yes	Unclear
Alreshidi et al., 2023 [15]	No	No	Yes	Yes	Yes	No	Unclear	Yes	Unclear
Surrati et al., 2023 [13]	Yes	Unclear	Yes	Yes	Yes	Yes	Yes	Yes	Unclear
Oraibi et al., 2024 [11]	No	No	Yes	Yes	Yes	Yes	Yes	Yes	Unclear
Al Hussaini et al., 2023 [12]	No	No	Yes	Yes	Yes	No	Unclear	Yes	Unclear
Al Zahrani et al., 2021 [18]	No	No	Yes	Yes	Yes	No	Unclear	Yes	Unclear
Hassounah, 2022 [16]	Yes	Yes	Yes	Yes	Yes	Yes	Yes	Yes	Unclear
Murad et al., 2020 [19]	No	No	Yes	Yes	Yes	No	Unclear	Yes	Unclear
Q1. Was the sample frame appropriate to address the target population? Q2. Were study participants sampled in an appropriate way? Q3. Was the sample size adequate? Q4. Were the study subjects and the setting described in detail? Q5. Was the data analysis conducted with sufficient coverage of the identified sample? Q6. Were valid methods used for the identification of the condition? Q7. Was the condition measured in a standard, reliable way for all participants? Q8. Was there an appropriate statistical analysis? Q9. Was the response rate adequate, and if not, was the low response rate managed appropriately?									

**Figure 2:**
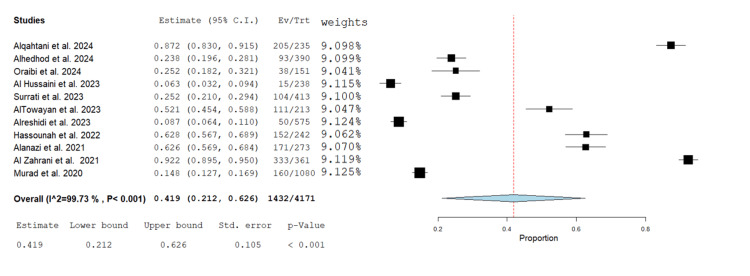
Forest plot of prevalence of HU among diabetic patients in Saudi Arabia HU: Hypoglycemia unawareness; Ev/Trt: Events per treated This plot displays the estimated prevalence and 95% CI for each included study (events/total), with study-specific weights on the right. The pooled prevalence is 41.9% (95% CI: 21.2% to 62.6%), based on a random-effects model; heterogeneity is very high (I² = 99.73 %, p < 0.001). Source studies: Alqahtani et al., 2024 [10]; Alhedhod et al., 2024 [6]; Oraibi et al., 2024 [11]; Al Hussaini et al., 2023 [12]; Surrati et al., 2023 [13]; AlTowayan et al., 2023 [14]; Alreshidi et al., 2023 [15]; Hassounah et al., 2022 [16]; Alanazi et al., 2021 [17]; Al Zahrani et al., 2021 [18]; Murad et al., 2020 [19]

This meta-analysis of 11 studies conducted between 2020 and 2024 demonstrates significant heterogeneity (I² = 99.73%, p < 0.001), with individual proportion estimates varying widely from 0.063 to 0.972. The overall pooled proportion is 0.419 (95% CI: 0.212-0.626), indicating a moderate prevalence or effect size, but there is considerable uncertainty (Figure 2).

**Figure 3:**
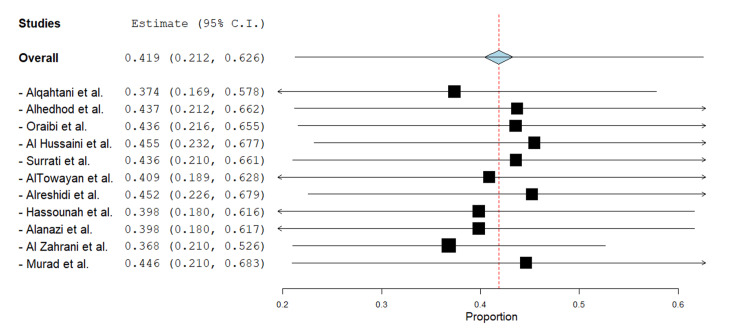
Leave-one-out sensitivity analysis of pooled proportion estimate This forest plot displays the pooled proportion estimate (0.419; 95% CI: 0.212–0.626) recalculated after sequentially omitting each study. Horizontal lines represent the leave-one-out 95% confidence intervals, and the red dashed vertical line marks the original pooled estimate, demonstrating the robustness of the overall result. Source studies: Alqahtani et al., 2024 [10]; Alhedhod et al., 2024 [6]; Oraibi et al., 2024 [11]; Al Hussaini et al., 2023 [12]; Surrati et al., 2023 [13]; AlTowayan et al., 2023 [14]; Alreshidi et al., 2023 [15]; Hassounah et al., 2022 [16]; Alanazi et al., 2021 [17]; Al Zahrani et al., 2021 [18]; Murad et al., 2020 [19]

This sensitivity analysis demonstrates the robustness of the pooled proportion estimate (0.419, 95% CI: 0.212-0.626) when individual studies are excluded one at a time. The recalculated estimates remain relatively stable, ranging from 0.368 (after excluding Al Zahrani et al.) to 0.455 (after excluding Al Hussaini et al.), all falling within moderate effect size boundaries (0.37-0.46). The minimal fluctuation in point estimates (less than ±0.05 from the overall estimate) and the consistently overlapping confidence intervals indicate that any single study does not disproportionately influence the primary result. This supports the reliability of the meta-analysis despite the presence of high heterogeneity (Figure 3).

Discussion

This review highlights that HU and IAH are common among individuals with diabetes in Saudi Arabia. However, the reported prevalence varies significantly depending on the assessment tool, population, and setting used. The pooled prevalence was 41.9% (95% CI: 21.2%-62.6%), showing high heterogeneity. Leave-one-out analyses indicated that while the results were numerically stable, the confidence intervals were consistently wide, suggesting that the findings are robust but cannot be summarized with a single estimate. Across the studies reviewed, factors such as longer diabetes duration, older age, female sex, lower socioeconomic status, and macrovascular comorbidities were frequently associated with HU/IAH. There are substantial gaps in knowledge and management regarding glucagon use, asymptomatic hypoglycemia, prevention strategies, and appropriate first-aid carbohydrates. These gaps are concerning given the significant risks of severe outcomes, including loss of consciousness and seizures.

In Asia, the incidence of IAH identified by the Gold-TW criteria among patients with insulin-treated T2DM was reported to be 19.6% in Singapore and 5.93% in Jordan. In contrast, the Clarke-TW criteria indicated that the prevalence of IAH was 13.7% in Singapore and 17.01% in Jordan [20,21]. Cheng et al. found that their results showed higher percentages compared to earlier studies, revealing that 41% of individuals with T2DM on insulin had IAH according to the Gold-TW criteria. In contrast, the prevalence was noted at 28.2% with the Clarke-TW criteria [22]. Additionally, a separate investigation conducted in Spain found that 12.0% of insulin-treated T2DM patients experienced IAH based on the Gold-TW criteria. In comparison, 10.2% were identified under the Clarke-TW criteria, indicating a strong correlation and good agreement between these two assessment methods [23].

In our review, reported IAH prevalence ranged from 14.8% to 20% in a nationwide online survey, depending on whether the Gold or Pedersen‑Bjergaard single-item method was used [19]. In a specialty center cohort using the Clarke method, 23.8% had IAH, with higher odds among people with T2DM in that setting [6]. In a mixed insulin-treated cohort from Madinah, the Clarke method yielded 25.2%. In comparison, the modified Pedersen‑Bjergaard scale nearly doubled that estimate to 48.9%, illustrating how instrument choice alone can shift prevalence bands [13]. Among adults with T2DM sampled online in Al‑Qassim, more than half screened positive by both Clarke (52.1%) and Gold (53.5%), contrasting sharply with nationwide estimates and suggesting selection and measurement effects in survey-based samples [14]. The T1DM-focused studies also diverged: a Jazan clinic cohort using Clarke reported 25.2% IAH [11], whereas a Riyadh center using Gold reported 62.8% [16]. Together, these patterns point to three drivers of heterogeneity: instrument (Clarke vs Gold vs Pedersen‑Bjergaard), sampling frame (clinic-based vs online), and case‑mix (T1DM vs T2DM proportions and insulin intensity) [6,11,13,14,16,19].

Moreover, knowledge surveys revealed wide dispersion, from predominantly poor awareness in Aseer and Al Jouf to largely “good” awareness in Al‑Ahsa, driven by different instruments and thresholds rather than true contrasts in competence [10, 12, 17]. Even in cohorts labeled “good,” critical deficits persisted: only one-third correctly understood glucagon administration, and fewer than half recognized asymptomatic hypoglycemia or driving-related risks [12]. Across regions, respondents commonly managed acute hypoglycemia with sweets or fruit juice. At the same time, preventive strategies emphasizing frequent self-monitoring were less prevalent, a pattern noted in Aseer, Al Jouf, and Jeddah [10,17,18]. Physician counseling and structured materials (pamphlets/booklets), as well as social media exposure, were associated with better knowledge scores, arguing for multimodal education strategies adapted to local information ecosystems [12,18]. Non-insulin-treated T2DM populations also showed modest awareness with frequent symptomatic episodes, indicating missed opportunities for education beyond insulin users [15]. Taken together, education quality appears pivotal, especially for domains with the highest stakes (glucagon, asymptomatic detection, prevention planning) [10,12,15,17,18].

Multiple factors contribute to the occurrence of IAH in individuals with T2DM. These include a history of severe hypoglycemic events within the past year, increased instances of hypoglycemia over the previous six months, lower levels of education, poor adherence to medication, and the presence of diabetes-related comorbidities [20,24]. In terms of duration, a study by Cheng et al. indicated that the prevalence of IAH was notably higher among those who had used insulin for less than a year compared to those who had been on insulin for five years or more, irrespective of the criteria applied [22]. Additionally, using insulin properly, by ensuring precise injection techniques, proper dosage, and timely administration, can significantly decrease the chances of experiencing hypoglycemia. Therefore, it is essential to educate individuals with T2DM about the correct methods of insulin delivery, as this knowledge can lower the risk of hypoglycemia and improve overall survival rates [25].

Furthermore, several studies explored predictors of IAH, but findings were inconsistent. While Van et al. reported higher prevalence among non-Caucasians, those on complex insulin regimens, and individuals with lower BMI or tighter glycemic control [26], other studies did not confirm associations with gender, age, or diabetes duration [23]. Instead, both Capre et al. and Schopman et al. emphasized the link between IAH and recent hypoglycemic history, suggesting that recurrent exposure may blunt symptom recognition [23,27]. Differences across studies may reflect variations in study populations, assessment tools, and definitions of IAH.

In our review, the duration of diabetes consistently aligned with higher IAH risk: ≥10 years in T1DM (Riyadh) and Jazan was associated with impaired awareness, and >15 years was associated with IAH in the nationwide survey [11,16,19]. Age and sex patterns mirrored international data; older age and female sex were associated with IAH in the nationwide study [19]. Socioeconomic gradients emerged in multiple cohorts: lower education and income were linked to poorer awareness/IAH or lower knowledge (Al Jouf, Jeddah, Aseer), while physician-delivered education and media exposure were associated with better scores (Al‑Ahsa) [10,12,17,18]. Higher BMI (≥25) and employment status were associated with IAH in Jazan, while more frequent glucose monitoring appeared protective, suggesting behavioral levers that can be targeted [11]. Associations by diabetes type were mixed. In Al‑Ahsa, T2DM had higher IAH odds than T1DM in an insulin-treated clinic population [6], whereas the nationwide study found greater IAH in T1DM [19]. The Madinah cohort showed no type-related difference by Clarke but slightly higher IAH in T2DM by the Pedersen‑Bjergaard scale [13]. These contradictions likely reflect instrument and sampling effects, different insulin exposures, and center-specific case‑mix. Cardiovascular disease signaled higher HU risk: stroke and ischemic heart disease were independent predictors in Madinah, highlighting a plausible interplay between autonomic neuropathy, cognitive factors, and hypoglycemia detection [13]. Insulin intensity did not show a consistent directionality; notably, a lower insulin dose predicted HU in Madinah, counterintuitive but potentially confounded by clinical frailty or prior dose reductions after hypoglycemia [13]. Overall, the most stable signals across settings were duration, age, education/income, and markers of suboptimal follow-up, with additional context-specific factors modifying risk [6,11,13,16-19].

Based on the findings of this review, it is recommended that healthcare systems in Saudi Arabia implement routine screening for IAH using standardized tools, such as the Clarke or Gold questionnaires, every three to six months for individuals with insulin-treated diabetes. This is particularly important for those who have had diabetes for a longer duration or who experience frequent hypoglycemic episodes. Educational interventions should focus on crucial topics, such as how to administer glucagon, recognize asymptomatic hypoglycemia, and properly use fast-acting carbohydrates. These programs should be anchored by clinicians and designed with culturally relevant materials, including pamphlets and social media campaigns. For high-risk patients, continuous glucose monitoring should be integrated into their care to help identify silent hypoglycemic episodes. Additionally, therapeutic plans should be customized to fit individual risk profiles, simplifying complex insulin regimens when possible and reinforcing proper injection techniques. Future research should employ multicenter, probability-based sampling designs with standardized definitions and validated instruments to improve generalizability and methodological rigor. Expanding outreach to rural and underserved populations will also be essential for obtaining representative prevalence estimates and designing inclusive prevention strategies.

This review is limited by its reliance on cross-sectional studies, which prevents causal inference and may introduce recall bias. Considerable heterogeneity was present due to the use of different HU assessment tools, including Clarke, Gold, Pedersen-Bjergaard, and other non-standardized questionnaires. Convenience sampling was predominant, limiting national generalizability, while reliance on self-reported measures increased the risk of misclassification. In addition, we did not perform a formal assessment of publication bias because our primary analysis was conducted using OpenMetaAnalyst [9], which does not provide this function. Therefore, the possibility of unpublished or non-English studies influencing the pooled estimates cannot be excluded.

To address these limitations, future research should employ multicenter, longitudinal cohorts with probability-based sampling to enhance representativeness. Standardized assessment using validated tools, ideally the Clarke questionnaire, supplemented by continuous glucose monitoring data would improve accuracy. Expanding recruitment to rural and underserved areas is also important for generating more representative prevalence estimates. Such efforts would provide stronger evidence to guide targeted educational and clinical interventions aimed at reducing the burden of hypoglycemia unawareness.

## Conclusions

Hypoglycemia unawareness is a widespread and clinically significant issue among individuals with diabetes in Saudi Arabia. Its occurrence is consistently linked to modifiable risk factors, including prolonged disease duration, limited socioeconomic resources, and substantial gaps in patient knowledge. The wide variation in reported prevalence across studies underscores methodological inconsistencies, reinforcing the need for standardized assessment tools and protocols. Deficits in diabetes education and inadequate clinical follow-up emerge as persistent contributors to impaired awareness. These findings support the urgent implementation of national guidelines that mandate routine screening for HU and promote structured, culturally tailored education programs. Such initiatives should emphasize symptom recognition, appropriate management strategies, and preventive behaviors. Future research should prioritize longitudinal designs, standardized instruments, and representative sampling to understand long-term outcomes better and evaluate the effectiveness of educational and technological interventions, particularly the role of continuous glucose monitoring in restoring hypoglycemia awareness and improving patient safety.
